# The Application of MR Spectroscopy and MR Perfusion in Cerebral Syphilitic Gumma: A Case Report

**DOI:** 10.3389/fnins.2020.544802

**Published:** 2020-10-21

**Authors:** Linyang Cui, Jie Liu, Wenjun Zhang, Zushan Xu, Hongjun Hou

**Affiliations:** Department of Radiology, Weihai Central Hospital, Weihai, China

**Keywords:** cerebral syphilitic gumma, brain tumor, magnetic resonance imaging, MR perfusion, MR spectroscopy cerebral syphilitic gumma

## Abstract

Cerebral syphilitic gumma is a rare disease and can be misdiagnosed as a common brain tumor when only conventional imaging results are adopted. Differentiating between syphilitic gumma and tumors may be achieved by applying advanced magnetic resonance (MR) techniques, such as MR spectroscopy and MR perfusion. However, the MR perfusion characteristics of cerebral syphilitic gumma have not been reported yet. Here, we report a case of cerebral syphilitic gumma in a 52-year-old woman and discuss the imaging features of conventional MR, MR spectroscopy, and MR perfusion. The results suggest that the application of MR spectroscopy and MR perfusion could provide additional information that contributes to the diagnosis of cerebral syphilitic gumma.

## Introduction

Neurosyphilis is a chronic infection in the central nervous system caused by *Treponema pallidum*. Cerebral syphilitic gumma is a rare subtype of neurosyphilis disease and was first reported by Botalli in 1563 (Oblu, 1975). This disease is usually occult in onset, with headache, nausea, and vomiting as the most common clinical manifestations. The typical magnetic resonance (MR) imaging features of cerebral syphilitic gumma either are isolated or consist of multiple intracranial lesions growing from the meninges, with irregular enhancement and surrounding edema, which may closely resemble those of some brain tumors. Because of its rarity in the clinic and non-specific manifestations and findings on neuroimaging, cerebral syphilitic gumma is often misdiagnosed as brain neoplasm, which requires surgical treatment. Therefore, it is necessary to find ways to correctly diagnose syphilitic gumma before starting therapy, since patients with syphilis respond well to penicillin. In recent years, functional MRI techniques, such as MR spectroscopy and perfusion MRI have brought additional value to the differential diagnosis between tumors and non-tumor lesions. Previously, MR spectroscopy findings of cerebral gumma have been reported (Ventura et al., 2012) and seem to be helpful in generating a differential diagnosis. However, to date, there have been no studies that demonstrate the diagnostic utility of perfusion MRI in cerebral syphilitic gumma. In the current study, we are the first to report on the perfusion MRI features of cerebral syphilitic gumma. More importantly, we found that MR spectroscopy and perfusion MRI can help improve the specificity and capability of diagnosing cerebral syphilitic gumma under appropriate clinical circumstances.

## Case Report

**FIGURE 1:**
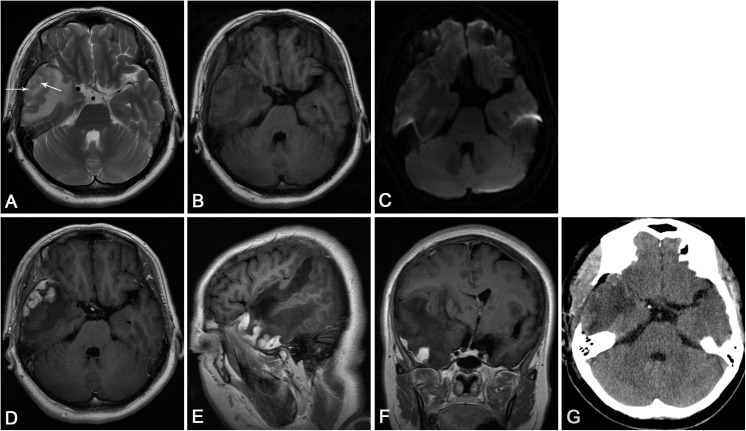
**(A)** Axial T2-weighted imaging showed multiple heterogeneous hypersignal nodules with central spots of hyposignal (arrows) in the right temporal lobe and significant edema around the lesion. **(B)** Axial T1-weighted imaging revealed the heterogeneous hyposignal nodules. **(C)** Diffusion-weighted imaging revealed slight hyperintense nodules. **(D)** Axial and **(E)** sagittal T1-weighted images with contrast demonstrated the enhancing nodules arising from the meninges, with significant perilesional edema. **(F)** Coronal T1-weighted images with contrast showed the mass effect of the nodules, which led to the compression of the right lateral ventricle and evident midline shift. **(G)** Post-operative CT showed that no nodules with a certain amount of hypodense edematous zone could be found on the right temporal lobe.

**FIGURE 2:**
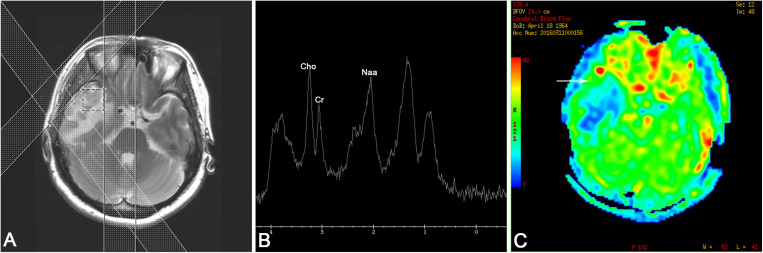
**(A)** Positioning image of single-voxel magnetic resonance (MR) spectroscopy. **(B)** MR spectroscopy [echo time (TE) 35 ms] revealed a slightly increased choline (Cho) peak and a moderate decrease in the peaks of creatine (Cre) and N-acetylaspartate (NAA). A lipid/lactate peak was observed at 0.9 and 1.3 ppm. **(C)** MR perfusion reflected that the cerebral blood flow of the nodules (arrow) was lower than that of the contralateral hemisphere, and significant low perfusion was observed in the edema area.

**FIGURE 3:**
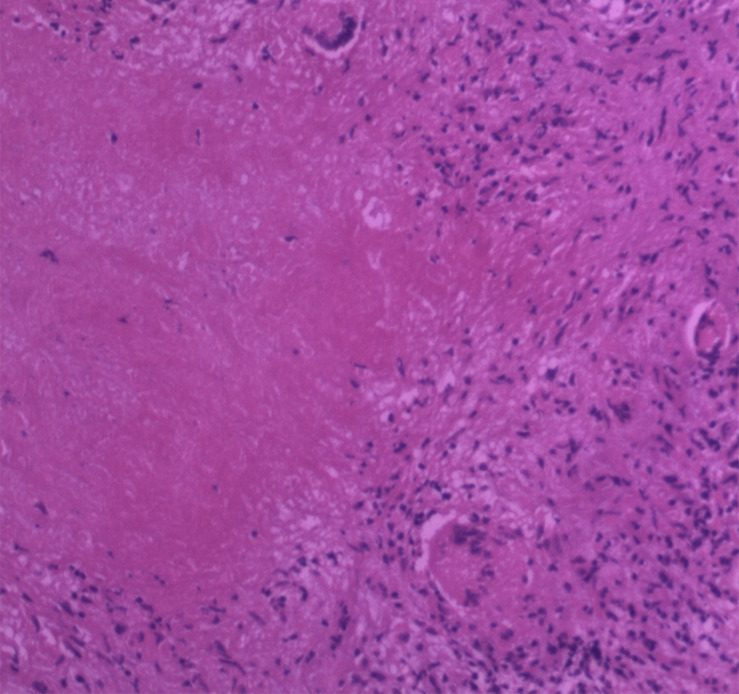
Histopathology showed granulation tissue with ischemic necrosis surrounded by multinucleated giant cells, plasmacytes, and lymphocytes (HE staining, original magnification ×100).

A 52-year-old woman was admitted with intermittent headache lasting for 5 months. The patient’s headache gradually worsened 1 week before admission, and vomiting occurred 2 days before hospitalization. Physical examination revealed normal findings. The patient had a history of hypertension and hyperlipidemia. No abnormality was found in the laboratory test results, which included blood routine examination, C-reactive protein, and erythrocyte sedimentation rate measurement. The serum tuberculosis antibody, toxoplasma antibody, and tumor markers were all negative. Serologic tests revealed positive results of the *T. pallidum* particle agglutination (TPPA) and toluidine red unheated serum test (TRUST), with a TRUST titer of 1:64. The human immunodeficiency virus (HIV) test was negative. Cerebrospinal fluid (CSF) examination was not performed. The patient denied any history related to venereal diseases, and the skin mucous membrane was free of rash and erythema. Conventional MR, proton MR spectroscopy, and perfusion MRI were carried out in the patient. All MR images were obtained using a 3.0 T clinical MR scanner (Discovery MR750, General Electric Healthcare, Milwaukee, WI, United States) with an eight-channel head coil. The conventional MRI sequences and parameters were as follows: axial T2-weighted imaging [repetition time (TR)/echo time (TE) = 5,200/90 ms, matrix = 512 × 512, field of view (FOV) = 24 cm, thickness = 5 mm, gap = 1.5 mm], axial T1-weighted imaging (TR/TE 1,750/25 ms, matrix = 320 × 256, FOV = 24 cm, thickness = 5 mm, gap = 1.5 mm), and diffusion-weighted imaging (TR/TE = 3,000/10 ms, matrix = 160 × 160, FOV = 24 cm, thickness = 5 mm, gap = 1.5 mm, b-value = 0, 1,000). Axial, sagittal, and coronal T1-weighted images after injection of gadolinium contrast agent (0.1 mmol/kg) were acquired. Perfusion imaging was performed by using a three-dimensional pseudo-continuous arterial spin labeling (ASL) technique with the specific imaging parameters: TR/TE = 4,600/10 ms, PLD = 1,525 ms, matrix = 128 × 128, FOV = 24 cm, thickness = 4 mm. ASL images were transferred to the workstation (ADW4.6, General Electric Healthcare) for post-processing and analyzed using FuncTool software (General Electric Healthcare) with the quantitative perfusion cerebral blood flow (CBF) map. Proton MR spectroscopy was obtained using a single-voxel point-resolved spectroscopy sequence (TR = 1,500 ms, TE = 35 ms). A volume of interest of 2 × 2 × 2 cm^3^ was selected from the lesion identified on the T2-weighted imaging sequence, and saturation bands were placed around the voxel. The process of shimming and water suppression was completed by the automatic pre-scanning program, making the bandwidth <7 and water suppression >97%. The MR machine’s individual configuration software was applied to complete the correction of the baseline, identification of each compound, and analysis of peak value and ratio of each compound. MRI (Figures 1A–C) revealed multiple nodules with evident perilesional edema in the right temporal lobe, which was characterized by hypointensity on T1-weighted images, mostly hyperintensity on T2-weighted images, and slight hyperintensity on diffusion-weighted imaging. Contrast-enhanced T1-weighted images (Figures 1D–F) showed significant enhancement of the nodules and adjacent meninges (“dural tail”). In addition, the right ventricle was compressed and the middle line structure was skewed to the left. Single-voxel MR spectroscopy with an echo time of 35 ms (Figures 2A,B) revealed a slightly increased choline (Cho) peak and a slight decrease in the peaks of creatine (Cr) and N-acetylaspartate (NAA). The Cho/Cr and Cho/NAA ratio over the lesion was 1.24 and 0.932, respectively. A peak rising between 0.9 and 1.3 ppm represented the lipid/lactate. Perfusion MRI (Figure 2C) suggested that regional CBF in the nodule area was lower than that of the contralateral normal regions, with the highest relative CBF value of 0.84 in the lesion area, while the edema area around the lesion presented less perfusion. Merely based on the clinical manifestations, laboratory results, and conventional MR images, the diagnosis of inflammatory granuloma should be first suspected, while a differential diagnosis including neoplastic lesions such as meningiomas and brain metastasis should also be considered. However, the findings of advanced techniques such as MR spectroscopy and MR perfusion provide more clues in diagnosing inflammatory granuloma and have effectively ruled out meningioma and brain metastasis. Considering the evident mass effect of the lesion, the patient underwent surgical resection of the nodules. During the surgery, multiple solid nodules were seen protruding from the dura of the temporal lobe into the adjacent cerebral cortex and had an unclear boundary, hard and brittle texture, and general blood supply. Severe edema in the brain tissue of the right temporal lobe was also observed. Post-operative pathology (Figure 3) revealed granulation tissue with ischemic necrosis surrounded by multinucleated giant cells, plasmacytes, and lymphocytes. Based on the above findings, the clinical diagnosis of cerebral syphilitic gumma was made. Penicillin treatment (18 million U/day) was given for 2 weeks after the operation, and the patient’s symptoms were relieved gradually. On the seventh day post-op, the patient was examined with non-contrast computed tomography (CT). The post-op CT (Figure 1G) showed no nodules, and a certain amount of hypodense edematous zone could be found on the right temporal lobe. After 6 months, the patient’s headache disappeared completely.

## Discussion

Since 2013, due to the AIDS epidemic and increase of the homosexual population and high-risk sexual behaviors, syphilis has made a comeback, affecting people of all ages and races (Puccio et al., 2019). Neurosyphilis accounts for 3.5% of syphilis cases with clinical or ophthalmologic manifestations (Ropper, 2019) and occurs mostly in untreated or undertreated patients. Neurosyphilis can occur during all stages of syphilis infection. According to the clinical manifestations, it can be divided into various forms, including asymptomatic, meningeal, meningovascular, parenchymal, and gummatous neurosyphilis. In 2016, Drago et al. (2016) reviewed 286 reported cases of neurosyphilis and found that the incidence of gumma was the lowest (3.5%) among all the subtypes. Cerebral syphilitic gumma is a benign proliferative lesion caused by the local meningovascular inflammatory response induced by *T. pallidum*, which generates a cell-mediated overreaction of the immune system. The pathological manifestation of syphilitic gumma is similar to that of tuberculosis, which includes inflammatory infiltration of a large number of lymphocytes and plasma cells and central caseous necrosis surrounded by epithelioid cells, multinucleated giant cells, and lymphocytes. Other major features include intimal hyperplasia and peripheral arterial inflammation.

Conventional MRI commonly reveals a single or multiple round mass located in the convex part of the hemisphere with varying degrees of edema. On T2-weighted images, the lesions were mostly displayed as hyperintense with the center having a low signal, while on T1-weighted images, the lesions were generally presented as a low signal, which showed annular enhancement under contrast conditions. The low signal on T2-weighted images indicates the caseous necrosis. In diffusion-weighted imaging, the non-caseating portion of the lesion presented a mildly elevated signal, which was probably due to the cytotoxic edema of inflammatory cells (Soares-Fernandes et al., 2007). Perilesional meningeal enhancement and thickening or a dural tail can be found in 35% of the cerebral syphilitic gumma cases (Fargen et al., 2009), while calcification or hemorrhage may occur in only a few cases. In our case, several typical imaging characteristics of syphilitic gumma were found in the multiple nodules in the right temporal cortex. The nodules showed a low signal on T1-weighted images, heterogeneous high signal on T2-weighted images, and mild hyperintensity on diffusion-weighted imaging, with enhanced presentation and enormous edema in the surrounding area under the contrast condition. Low signal foci on T2-weighted images and the characteristic “dural tail” were also seen. However, it was still difficult to differentiate between the diagnosis of cerebral syphilitic gumma and intracranial neoplasias due to the lack of specific MR characteristics. In clinical practice, cerebral syphilitic gumma is most commonly misdiagnosed as high-grade gliomas, metastatic tumors, or meningiomas, which leads to subsequent surgeries in the patients (Noel et al., 2011; Xia et al., 2017; Zhang et al., 2017; Li et al., 2019; Weng et al., 2019).

Advanced MRI techniques, such as MR spectroscopy and perfusion MRI, can greatly contribute to the differential diagnosis of neoplastic and non-neoplastic lesions by providing functional information. MR spectroscopy can reflect the change in cerebral metabolism and biochemistry from an objective perspective, including neuronal injury, membrane phospholipid metabolism, lipid storage, and energy and oxidative metabolism. High-grade glioma, metastatic tumor, and meningioma often exhibit prominent elevation of Cho and significantly decreased levels or even lack of Cr and NAA, while non-neoplastic lesions generally present slightly decreased levels of Cho, Cr, and NAA (Möller-Hartmann et al., 2002). The most frequently used chemical ratios to differentiate tumors from non-neoplastic diseases with MR spectroscopy are Cho/Cr and Cho/NAA (Callot et al., 2008), though there is little consensus in the literature regarding the actual integral value of Cho/Cr and Cho/NAA ratios. In general, a Cho/NAA ratio greater than 1 is considered to indicate a neoplasm (Callot et al., 2008). However, in a previous report, a Cho/Cr ratio of 1.97 or greater could indicate high sensitivity and specificity in differentiating between inflammatory lesions and tumors (Ferraz-Filho et al., 2009). Furthermore, the Cho/Cr and Cho/NAA ratios are higher in neoplastic diseases such as high-grade glioma, metastatic tumor, and meningioma. Perfusion MRI is an emerging technique that provides tissue hemodynamic results. ASL is a non-invasive perfusion imaging technique that has rapidly developed in clinical practice and can quantitatively detect the CBF by labeling the arterial blood as an inner tracer, thus obviating exogenous contrast. Previous research that explored the application of ASL in the evaluation of the flow perfusion of cerebral tumors indicated a high CBF value in high-grade gliomas, metastatic tumors, and meningiomas, which was speculated to be due to tumor angiogenesis (Soni et al., 2018; Huang et al., 2019). However, a few reports that focused on the perfusion MRI characteristics among infection cases indicated that, except for herpes simplex virus infection, the vast majority presented low perfusion due to lack of angiogenesis (Noguchi et al., 2016). In imaging with addition of contrast agents, the infection showed significant enhancement because of the destruction of the blood–brain barrier rather than an increase in blood supply. The MR spectroscopic findings in our case showed a mild increase in Cho as well as slight decreased Cr and NAA (Cho/Cr = 1.24; Cho/NAA = 0.932), which was similar to the observation in a previously published case report of intracranial syphilitic gumma (Ventura et al., 2012). In addition, the focal MR perfusion of the lesion in our case had a lower CBF compared to the contralateral normal brain tissue. The above functional MR results may contribute to the exclusion of common intracranial neoplasia, such as high-grade glioma, metastatic tumors, and meningioma in the differential diagnosis, thus further confirming the diagnosis of syphilitic gumma based on serologic tests and conventional MR findings. To our knowledge, this is the first report evaluating the perfusion MRI characteristics of cerebral syphilitic gumma.

Surgery should not be performed in cerebral syphilitic gumma patients without significant intracranial hypertension or progressive exacerbation of symptoms, while the intravenous administration of penicillin G for 2 weeks (18–24 million U/day) is recommended as treatment according to international guidelines.

It is generally accepted that the diagnosis of cerebral syphilitic gumma should be made based on the patient history, clinical manifestations, laboratory tests, and MRI results. However, non-specific symptoms and the inability of the patient to provide a medical history or concealing it may hamper diagnostic accuracy. Syphilis serology and CSF tests (e.g., TPPA, TRUST) are critical to the diagnosis. Although conventional MRI can provide clues for the diagnosis of cerebral syphilitic gumma based on certain characteristics, it is often misdiagnosed as high-grade glioma, metastatic tumors, and meningioma due to the lack of specific imaging features, which can be complemented by advanced MRI techniques. Combined application of conventional and advanced MRI and laboratory tests could increase the accuracy of the preoperative diagnosis of cerebral syphilitic gumma, thus avoiding unnecessary surgeries.

## Ethics Statement

Written informed consent was obtained from the individual for the publication of this case report, including any potentially identifiable images or data included in this article.

## Author Contributions

LC wrote the manuscript. WZ and ZX collected the patient’s materials. HH and JL guided the completion of this article. All authors contributed to the article and approved the submitted version.

## Conflict of Interest

The authors declare that the research was conducted in the absence of any commercial or financial relationships that could be construed as a potential conflict of interest.

## References

[B1] Callot V. Galanaud D. Le Fur Y. Confort-Gouny S. Ranjeva J. P. Cozzone P. J. (2008). (1)H MR spectroscopy of human brain tumours: a practical approach. *Eur. J. Radiol.* 67 268–274. 10.1016/j.ejrad.2008.02.036 18406554

[B2] Drago F. Merlo G. Ciccarese G. Agnoletti A. F. Cozzani E. Rebora A. (2016). Changes in neurosyphilis presentation: a survey on 286 patients. *J. Eur. Acad. Dermatol. Venereol.* 30 1886–1900. 10.1111/jdv.13753 27306850

[B3] Fargen K. M. Alvernia J. E. Lin C. S. Melgar M. (2009). Cerebral syphilitic gummata: a case presentation and analysis of 156 reported cases. *Neurosurgery* 64 568–576. 10.1227/01.NEU.0000337079.12137.89 19240620

[B4] Ferraz-Filho J. R. Santana-Netto P. V. Rocha-Filho J. A. Sgnolf A. Mauad F. Sanches R. A. (2009). Application of magnetic resonance spectroscopy in the differentiation of high-grade brain neoplasm and inflammatory brain lesions. *Arq. Neuropsiquiatr.* 67 250–253. 10.1590/s0004-282x2009000200014 19547817

[B5] Huang R. Y. Bi W. L. Griffith B. Kaufmann T. J. Fougère C. Schmidt N. (2019). Imaging and diagnostic advances for intracranial meningiomas. *Neuro Oncol.* 21(Suppl._1), i44–i61. 10.1093/neuonc/noy143 30649491 PMC6347083

[B6] Li C. Wang S. J. Tang G. C. Liu L. T. Chen G. X. (2019). Neuroimaging findings of cerebral syphilitic gumma. *Exp. Ther. Med.* 18 4185–4192. 10.3892/etm.2019.8089 31772624 PMC6861868

[B7] Möller-Hartmann W. Herminghaus S. Krings T. Marquardt G. Lanfermann H. Pilatus U. (2002). Clinical application of proton magnetic resonance spectroscopy in the diagnosis of intracranial mass lesions. *Neuroradiology* 44 371–381. 10.1007/s00234-001-0760-0 12012120

[B8] Noel C. B. Moeketsi K. Kies B. (2011). Cavernous sinus syndrome, an atypical presentation of tertiary syphilis: case report and review of the literature. *Clin. Neurol. Neurosurg.* 113 65–67. 10.1016/j.clineuro.2010.08.007 20884116

[B9] Noguchi T. Yakushiji Y. Nishihara M. Togao O. Yamashita K. Kikuchi K. (2016). Arterial spin-labeling in central nervous system infection. *Magn. Reson. Med. Sci.* 15 386–394. 10.2463/mrms.mp.2015-0140 27001393 PMC5608113

[B10] Oblu N. (1975). “Gumma of the brain,” in *Handbook of Clinical Neurology [M]*, eds Vinken P. J. Bruyn G. W. (New York, NY: Elsevier), 427–434.

[B11] Puccio J. A. Cannon A. Derasari K. Friend R. (2019). Resurgence of syphilis. *Adv. Pediatr.* 66 231–244. 10.1016/j.yapd.2019.03.006 31230696

[B12] Ropper A. H. (2019). Neurosyphilis. *N. Engl. J. Med.* 381 1358–1363. 10.1056/NEJMra1906228 31577877

[B13] Soares-Fernandes J. P. Ribeiro M. Maré R. Magalhães Z. Lourenço E. Rocha J. F. (2007). Diffusion-weighted magnetic resonance imaging findings in a patient with cerebral syphilitic gumma. *J. Comput. Assist. Tomogr.* 31 592–594. 10.1097/01.rct.0000284391.91320.89 17882038

[B14] Soni N. Srindharan K. Kumar S. Mishra P. Bathla G. Kalita J. (2018). Arterial spin labeling perfusion: prospective MR imaging in differentiating neoplastic from non-neoplastic intra-axial brain lesions. *Neuroradiol. J.* 31 544–553. 10.1177/1971400918783058 29890916 PMC6243465

[B15] Ventura N. Cannelas R. Bizzo B. Gasparetto E. L. (2012). Intracranial syphilitic gumma mimicking a brain stem glioma. *AJNR Am. J. Neuroradiol.* 33 E110–E111. 10.3174/ajnr.A3236 22723064 PMC7965502

[B16] Weng C. Huang K. Jiang T. Zhou G. Wu T. (2019). Cerebral syphilitic gumma masquerading as cerebral metastatic tumors: case report. *Neurosurg. Focus* 47:E15. 10.3171/2019.5.FOCUS1953 31370018

[B17] Xia D. Y. Zhu M. F. Liu C. G. Dai Y. Li Z. B. Jiang X. C. (2017). Cerebral syphilitic gumma misdiagnosed as a malignant brain tumor. *J. Craniofac. Surg.* 28 e170–e172. 10.1097/SCS.0000000000003191 27755440

[B18] Zhang L. L. Zhou Y. L. Chen J. Yan W. L. Kong Q. T. Chen P. (2017). A case of a cerebral syphilitic gumma developed in a few months mimicking a brain tumor in a human immunodeficiency virus-negative patient. *Br. J. Neurosurg.* 31 481–483. 10.3109/02688697.2016.1173190 27088540

